# Myoma Uteri and Its Treatment

**Published:** 1894-06-02

**Authors:** 


### PROGRESS IN GYNÆCOLOGY.

### MYOMA UTERI AND ITS TREATMENT.

A. F. Currier,[1] after reviewing some of the methods pursued in the treatment of uterine fibroids—*e.g.*, Schrœder's enucleation of interstitial growths, the galvanic treatment, and that usually adopted by Ergot—approaches the practical question "as to the advisability of subjecting patients to the risks of a severe and possibly fatal operation, together with the mutilation attending the removal of an important organ." The statement, generally attributed to Keith, that women suffering from a uterine fibroid have never succumbed to the direct effects of such a tumour should not be taken too literally. It is certain that the constant loss of blood, the pain, discomfort, and disturbance of several important functions have impaired the lives of many women, and finally brought them to a premature grave. The circumstances, the surroundings, and the nature of every female suffering from a myoma must be taken into account when considering the question of operation. The effect of the tumour upon the nervous system, and the anxiety as to the possible result is more pronounced in some patients than in others. The rate of development of each tumour is also an important factor in considering the treatment to be adopted. Kleinwächter, Gusserow, and Schorler have specially studied this subject. According to Kleinwächter the rate of growth in most cases is slow, more so in the fibroid variety than in the true myoma. Temporary enlargement is frequently found during menstruation and pregnancy, and temporary diminution is sometimes found during and after a debilitating illness. The diminution and final disappearance of the tumour does not in all cases follow, as was at one time supposed, the occurrence of the menopause; and in some cases the growth may even increase in size. The author classifies the methods of treatment as (1) palliative, (2) semi-radical, (3) thoroughly radical.

The chief among the palliative methods is galvanism, which almost always will relieve pain and hæmorrhage, though Dr. Currier has never found that the size of the tumour has been reduced by this method of treatment. Puncture of the tumour by one or other of the electrodes the author has never attempted, as he considers it too dangerous. Electrical treatment, however, should not be continued for long if beneficial results do not follow after a reasonable time. Harm may arise from long continued use, and militate against successful surgical operations. Treatment by ergot, hydrastis canadensis, and other drugs is well known and needs no comment, as their advantages and disadvantages have been frequently discussed. The author does not regard diet as an important factor in the treatment of these cases, as any course of diet which may affect the tumour beneficially must impair the general health too greatly. Curettage of the endometreum may be successfully employed when the hæmorrhage is excessive, but its effects are soon lost as the endometreum is renewed.

Semi-radical treatment has for its object the premature induction of the climacteric. For this purpose abdominal section is performed, and includes removal of the ovaries and tubes or ligature of the uterine and ovarian arteries, or a combination of the two. Unfortunately this method is not always successful in checking the hæmorrhage or causing diminution in the growth of the tumour on account of the extensive collateral circulation, but it is indicated in those cases in which there is (1) obstinate dysmenorrhœa, (2) where a small myomatous growth exists, with much hæmorrhage and pain, (3) where the growth is large and its complete removal is extra hazardous, and (4) when the patient is too weak to permit of total extirpation. The author's experience of the semi-radical method of operation is in favour of it, as the uterus and tumours have generally undergone atrophy in the course of a year.

The radical method of treatment consists in the removal of the entire tumour, or of the removal of the tumour with the uterus and appendages. In this class sub-peritoneal tumours can be successfully removed, especially if their pedicles be small, without impairing the structure of the uterus. Care, however, must be taken to ligature securely all the vessels and to unite the edges of the divided peritoneum over the uterine wound. For all other uterine fibro-myomata complete extirpation of the growth with the uterus and tubes is indicated. When complete extirpation is practised the treatment of the stump is a most important point, and the author advocates the intra-peritoneal method of treatment of the stump, as practised by Chrobak, Dudley, and others. The extra-peritoneal method of treatment of the stump he considers as decidedly clumsy, though it has the advantage of keeping any septic mischief within reach; an advantage, however, which is counterbalanced by many disadvantages. For the radical cure of fibroids, the author specially recommends a modification of the operation described years ago by Freund for the removal of the uterus for cancer. The improvement made in the technique of the operation, together with the position of the patient, as recommended by Trendelenberg, have rendered the operation for total extirpation of the uterus safer and easier than formerly, while the shock which accompanies the operation is less. By this method, not only is the entire uterus removed with its myoma, but the broad ligaments which are carried down into the vagina act as drains, and so favour the escape of any collection of septic matter which may accumulate, and which formerly was the chief source of danger.

### Electrical Treatment of Extra-Uterine Pregnancy.

A. Brothers[2] has collected eighty-five cases of extra-uterine pregnancy treated by electricity, and believes that there are others which have been treated in a similar manner, not included in this list. The majority of the cases in this table have occurred in America, but this method has also been tried in England five times, in Russia five times, and once in Italy, Canada, France and Australia. Out of the eighty-five cases death resulted in six, but this result in three cases could not be ascribed to the electrical treatment, for it had already been abandoned in favour of aspiration or abdominal section. Galvano-puncture was resorted to in six cases, faradism in forty-three cases, and galvanism in thirty-six cases. In several instances the galvanic or faradic current were used in the same case. The average current strength is not stated. The author is convinced of the efficacy of this method of treatment in suitable cases, especially in the early stages of the complaint, and maintains that, in spite of all that has been asserted to the contrary, the treatment will continue to prove simple and successful.

### Comparative Microscopical Studies of the Ovaries.

Francis Fosster[3] reaches the following conclusions on small cystic degeneration of the ovaries: (1) Small cysts are of common occurrence, not only in human ovaries, but also in those of the sow, ewe, and cow. (2) Small cysts are originally Graafian follicles. He thus confirms the researches of Nagel. (3) In a process not exactly pathological the stratified lining spithelium of the Graafian follicle undergoes peculiar changes leading to its disappearance. (4) The epithelium first breaks up into an indifferent or medullary tissue, and from this arises myxomatous vascularized connective tissue. (5) The type of the newly-formed myxomatous tissue varies in different animals. (6) The newly-formed myxomatous tissue is scantily supplied with blood vessels, which probably grow into it from without. (7) The myxomatous lining of the cyst wall is always well defined toward the outer fibrous coat. (8) The ovale is present at the beginning of the formation of a small cyst; later it probably perishes, owing to the changed environments.

### Associated Parovarian and Vaginal Cysts.

Details of three cases were read by Amand Routh before the Obstetrical Society of London. The cysts originated in Gartner's ducts, and were formed by the distension of the vaginal and broad ligament portions of that duct. It was suggested that those cases of profuse watery discharge from the vagina, not coming from the bladder or uterus, might originate from this source. Whenever the whole duct is distended the best treatment to adopt is to lay open freely the vaginal portion as far as the base of the broad ligament, and to encourage the supra-vaginal portion to contract and close up. From the evidence afforded by these three cases the author concludes that (1) Gartner's duct can be traced in some cases in the adult female from the parovarium to the vestibulum valve, ending just beneath and to one side of the urethral orifice. (2) Homology tends to show that many of Schüller's glands are diverticula of Gartner's ducts, just as the vesiculæ seminales are diverticulæ of the vasa differentia. (3) Gartner's duct, if patent, may become distended at any part of its course, constituting a variety of parovarian cyst if the distension be in the broad ligament portion, and a vaginal cyst if the distension be in the vaginal portion.

### Treatment of Hæmato-Salpinx Due to Genital Atresia.

In the treatment of the above condition Meyer[5] formulates the following rules for operation: (1) When hæmato-salpinx is recognised, especially where the atresia is situated high up in the genital tract. (2) In cases in which the distension of the tubes is found in patients with congenital atresia who have severe disturbances during the menstrual period. (3) When, after evacuating, retained menstrual blood tumours can still be made out which may be regarded as distended tubes that cannot discharge their contents into the uterus. (4) If only one tube be affected it is better to remove both in order to prevent future trouble. (5) Supra-vaginal amputation of the affected uterus is an unnecessarily severe operation.

[1] Amer. Journ. Obstet., Jan., 1894, p. 19. [2] Amer. Journ. Obstet., Jan., 1894, p. 56. [3] Amer. Journ. Obstet., Feb., 1894, p. 145. [4] Obstet. Soc. of Lond., Trans. April, 1894, and Lancet, April 14, 1894, p. 935. [5] Deutsche Med. Wochenschrift, 1893, No. 39.

